# The relationship between farmers’ entrepreneurial behavior and macroeconomics based on the probit regression model and entrepreneurial psychological capital

**DOI:** 10.3389/fpsyg.2022.954874

**Published:** 2022-09-23

**Authors:** Hao Li, Na Qi, Zheng Li, Wanying Ma

**Affiliations:** ^1^Rural Development Institute, Chinese Academy of Social Sciences, Beijing, China; ^2^School of Philosophy and Sociology, Jilin University, Changchun, China; ^3^Department of Life Culture, Beijing College of Social Administration, Beijing, China; ^4^College of Finance and Economics, Shanghai Lida University, Shanghai, China; ^5^College of Business, Changchun Guanghua University, Changchun, China

**Keywords:** entrepreneurial psychological capital, farmers’ entrepreneurial behavior, questionnaire survey, macroeconomics, probit regression model

## Abstract

At present, the research on the influence mechanism of psychological capital on farmers’ entrepreneurial behavior is relatively mature. However, the relationship between farmers’ entrepreneurial behavior and macroeconomics by entrepreneurial psychological capital (PsyCap) is still unclear. Based on this, firstly, this work analyzes the entrepreneurial PsyCap in detail. Secondly, the research hypothesis is put forward and a conceptual model is implemented. A questionnaire is designed to analyze the current situation of farmers’ entrepreneurial PsyCap and entrepreneurial behavior. Finally, a structural equation model (SEM) is implemented to explore the relationship between farmers’ entrepreneurial behavior and macroeconomics. The path test of the SEM is utilized to obtain the following. Macroeconomic growth has a significant positive impact on entrepreneurial behavior. Macroeconomics can affect farmers’ entrepreneurial behavior to varying degrees by affecting the four entrepreneurial PsyCap of farmers’ subjective cognition, Tenacity, hope and open-mindedness. This indicates that entrepreneurial PsyCap plays an intermediary role between farmers’ entrepreneurial behavior and macroeconomics. The purpose of this work is to explore the relationship among farmers’ entrepreneurial behavior, macroeconomics, and the role of entrepreneurial PsyCap through empirical analysis, thereby providing a theoretical reference for the subsequent country’s optimization of farmers’ entrepreneurial strategies.

## Introduction

As an important part of nationwide enterprise-starting, farmers’ entrepreneurship is not only a vital way to improve the income level of farmers, but also a momentous measure to promote the revitalization of rural talents. The entrepreneurial enthusiasm of farmers has been fully stimulated, which will help to facilitate the transformation of rural production and lifestyle. To guide elite talents to enter the countryside, encourage farmer entrepreneurs to grow and develop, realize the dual-wheel drive of urbanization and industrialization, and stimulate the vitality of rural economic development, scientifically accelerate the sustainable development of the rural economy is inseparable from rural innovation and entrepreneurship. Therefore, the discussion of rural innovation and entrepreneurship will be a critical topic in future rural research. In recent years, with the mushroom growth of the economy, the shortage of social jobs or the saturation of post personnel has made the efficiency of rural migrant work lower, and more and more farmers choose to start their own businesses because they are not satisfied with the status quo of migrant work (Cross and Swart, 2021). However, with the continuous development of macroeconomics, the success rate of farmers’ entrepreneurship is also affected (Kobba et al., 2021). There is an appreciable positive correlation between macroeconomics and the formation of farmers’ entrepreneurial behavior.

From the perspective of the initial source of farmers’ psychological capital (PsyCap), the macro-entrepreneurship environment has a greater impact on their development (Yueh et al., 2020). A good and stable political and social environment helps farmers to increase their hope, self-confidence, and optimism, and a relaxed economic environment helps farmers to improve their resilience (Schneider, 2022). On the contrary, a harsh and uncertain environment may lead to a decline in the level of PsyCap of farmers, and even lead them to develop negative psychology and choose negative behaviors, such as depression, suicide, absconding with money, etc. (Shi and Wang, 2021). PsyCap is the psychological reflection of people on something, and people make a series of corresponding behaviors by establishing their own entrepreneurial PsyCap. Hence, it is a momentous factor to further people’s own development. Some studies have found that the personal entrepreneurial PsyCap of farmers has a vital influence on forming their entrepreneurial awareness (Feng and Chen, 2020). Entrepreneurial PsyCap can have a significant indirect influence on farmers’ entrepreneurial behavior through traditional financial, human, and social capital (Tma et al., 2020). Entrepreneurial guidance, optimism, and self-efficacy are positively correlated with farmers’ entrepreneurial behavior (Zhong et al., 2020). Moreover, entrepreneurial guidance affects entrepreneurial intention by means of optimism and self-efficacy, and it plays a mediating role between entrepreneurial guidance and entrepreneurial intention (Ephrem et al., 2021).

According to relevant literature, it can be found that there are still some problems with the relationship between macroeconomics and farmers’ entrepreneurial behavior, which is mainly reflected in the fact that the current research only explores the impact mechanism of PsyCap on farmers’ entrepreneurial behavior. It has not been proved whether the relationship between macroeconomics and farmers’ entrepreneurial behavior and the role of entrepreneurial PsyCap have an influence in this process. According to this, firstly, a detailed analysis of entrepreneurial PsyCap is carried out. Secondly, the research hypotheses are proposed and a conceptual model is implemented. Meanwhile, a questionnaire is designed to analyze the current status of farmers’ entrepreneurial PsyCap and entrepreneurial behavior. Finally, a structural equation model (SEM) with entrepreneurial PsyCap as an intermediary variable is constructed to explore the relationship between macroeconomics and farmers’ entrepreneurial behavior. The innovation lies in using the probit regression model to test the robustness of the questionnaire data, aiming to explore the relationship between farmers’ entrepreneurial behavior and macroeconomics (Novanda et al., 2021). The purpose of this work is to use the relationship among farmers’ entrepreneurial behavior, entrepreneurial psychology, and macroeconomics, to provide direction and reference for a better grasp and efficient control of farmers’ entrepreneurial phenomenon and macro-economy.

## Theoretical and hypothesis

### Related concepts

#### Farmers’ entrepreneurial behavior

Farmers’ entrepreneurial behavior refers to the process in which farmers with certain entrepreneurial ability and certain entrepreneurial capital maximize benefits by distributing and combining various production factor resources, further innovating the form of organization and management, and increasing labor employment on the basis of discovering and utilizing market space (Chen et al., 2020). Under the influence of internal and external environment and other factors, farmers’ entrepreneurial behavior is the use of their own experience, technology, capital, and other capabilities to identify and utilize entrepreneurial opportunities to carry out independent economic activities, or a social phenomenon of engaging in large-scale and characteristic agricultural production and operation activities. Entrepreneurship for farmers is a complicated process (Rogoza et al., 2018). On the one hand, farmer entrepreneurs will fully consider their own comprehensive quality, economic resources, ability to obtain information, and social relations they have in light of their specific circumstances. On the other hand, affected by their own entrepreneurial motivation, farmers identify and grasp entrepreneurial opportunities, and finally make the choice of entrepreneurial behavior and whether to carry out actual entrepreneurial activities through a comprehensive assessment of their risk resistance (Janker et al., 2021).

According to the research literature, there are five factors that drive farmers to start their own businesses:

(1) To earn a living: At this stage, land resources are reduced, farmers’ lives are affected, and some farmers have embarked on the road of entrepreneurship under the pressure of resources and the environment;

(2) To adapt to the development of the times: With the continuous development of society, people’s various concepts have changed, leading to some farmers wanting to adapt to the times through entrepreneurship, to create a happy life;

(3) To stimulate independence: some farmers have accumulated a lot of experience in the process of working, thereby stimulating their own independent creativity, so they take the road of entrepreneurship;

(4) For self-esteem: Some farmers are unwilling to live a mediocre life and want to further enhance their own value through entrepreneurship;

(5) To achieve life goals: Some farmers have new life goals during the process of working, but due to the current situation of working, they choose to start a business to achieve life goals, to obtain higher life value.

#### Macroeconomics

Macroeconomic means the economy at the macro level, which is also referred to as aggregate economic activity. It mainly expresses the entire national economy or the overall national economy, its economic activities and operating statuses, such as total supply and total demand; the total value of the national economy and its growth rate; the main proportional relationship in the national economy; the overall level of prices; the total level of labor and employment and unemployment rate; the total size and growth rate of currency issuance; the total size of import and export trade and its changes, etc. (Chen, 2019). Macroeconomics stands for theoretical knowledge about it.

#### Entrepreneurial psychology

**Figure 1:**
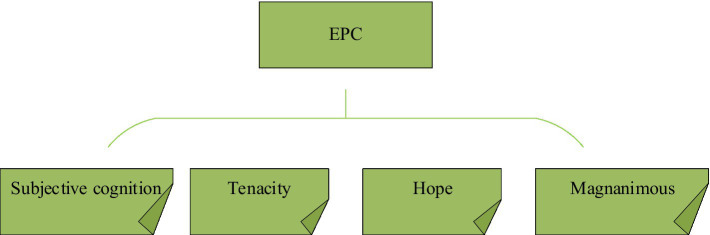
The dimension of EPC.

Traditional economic capital, human capital, and social capital are prominent factors that affect people’s entrepreneurship (Lv et al., 2021). However, with the rapid extension of society and the increasingly complex entrepreneurial environment, entrepreneurs often face a shortage of capital, and human and social resources and need to make effective strategic decisions quickly. The psychology of entrepreneurs is particularly key (Schneider and Saeed, 2021). In response to this phenomenon, scholars put forward entrepreneurial PsyCap on the basis of the original economic capital, human capital, and social capital. Educational Psychology Counseling (EPC) represents the sum of psychological resources that can meet the emotional requirements in the entrepreneurial process and advance entrepreneurial success. It mainly comprises four dimensions (Can, 2021). The dimension of EPC is displayed in Figure 1.

Subjective cognition mainly illustrates people’s subjective speculation and judgment on whether they can successfully carry out a certain action, which consists of two components: outcome expectations and efficacy expectations. Outcome expectations are judgments of the possible consequences of a particular action in a particular situation. For example, farmers’ speculation about whether they can start a business successfully. Efficacy expectations are beliefs about their ability to achieve a certain behavior, for instance, farmers’ subjective judgments about their ability to successfully start a business. Self-efficacy, as the core of an individual’s subjective factor, affects people’s physical and mental response to situations such as risk, failure, and stress, and determines an individual’s entrepreneurial behavior and even entrepreneurial success or failure (Cumming et al., 2021).

Tenacity mainly indicates the ability of people to adjust themselves as soon as possible, recover well, and resolve difficulties when faced with stress and setbacks (Santoro et al., 2020). Farmer entrepreneurs with this personality trait can remain rational when faced with risks, objectively analyze, take precautions and make predictions; when encountering obstacles, they can also try their best to find solutions and make full use of existing resources to remove obstacles (Rosli et al., 2021). It can be seen that tenacity is the driving force for farmers to form entrepreneurial awareness and dare to start a business.

Hope means that people make plans and goals for an event, and even if they face adversity or face great obstacles, they will persevere in moving toward the goal (Ji et al., 2021). In the process of starting a business, hope is a sense of belief for people, so that entrepreneurs continue to challenge themselves and strive to overcome various difficulties until they achieve their goals. Entrepreneurs can gain more hope from successive successful experiences and become more active, and these accumulated hope experiences will become stable entrepreneurial PsyCap for farmer entrepreneurs (Wijaya et al., 2020).

Open-mindedness stands for people’s positive expectations for future events, and is an attitude towards life that accepts and strives (Gao et al., 2020). When there is a setback, optimists see it as temporary, caused by external factors, and limited to the here and now. As a result, optimists do not give up self-belief and choose to keep trying (Pathak and Goltz, 2021). In the process of starting a business, entrepreneurs with optimistic personalities can quickly get rid of negative psychology, respond quickly to problems, and make events develop in the direction they expect (Christy et al., 2021).

### Proposed structural model

#### Macroeconomics and farmers’ entrepreneurial behavior

Regarding the relationship between macroeconomics and entrepreneurship, some scholars have observed the relationship between the economic cycle and entrepreneurial activities at the macro level, while others have devoted themselves to exploring entrepreneurial models and discovered the predictive role of some external environmental variables, and related to macroeconomic indicators (Yu and Xu, 2022). This study refers that macroeconomic regulation can drive people’s enthusiasm for starting a business, and people will provide certain conditions for starting a business with the growth of macroeconomics and their own actual conditions. And, with the support of this entrepreneurial condition, a series of entrepreneurial behaviors are formed (Yamamura and Lassalle, 2022). In addition, previous studies have also shown that economic recession can have a significant impact on entrepreneurial intentions and behavior, and it also affects entrepreneurial psychology (Yang et al., 2021). At the macro level, a number of studies using cross-country panel data denote that rising unemployment during recessions leads to an increase in overall entrepreneurial activity, mostly low-cost entrepreneurship that provides low-cost goods or services. Entrepreneurship is a way for individuals to meet their survival needs when they lack other sources of employment, and many self-employments without employees falls into this type (Anisa et al., 2021). Moreover, although an economic recession can bring entrepreneurial advantages such as high availability of human capital and second-hand resources, compared with the economic boom period, there are a series of obstacles such as low market demand and low expected returns. That is, there are various business opportunities under different macroeconomic conditions (Bandyopadhyay et al., 2021). Compared with self-employment entrepreneurship based on survival motives, the hiring of others is more driven by opportunity. Thus, when it occurs often depends on the individual’s ability to utilize diverse business opportunities.

From this, the following hypotheses are made:

*H1*: Macroeconomics has a prominent positive impact on the formation of farmers' entrepreneurial behavior.

#### Macroeconomics and EPC

Entrepreneurial PsyCap will be affected by various factors. Among them, personal factors are the most internal and stable source of influence on it; macroeconomics is an important external factor affecting individual entrepreneurial PsyCap (Ullah et al., 2020). Studies have indicated that macroeconomics affects farmers’ entrepreneurial psychology to a certain extent.

In the manner of this, the following hypotheses are put forward:

*H2a*: Macroeconomics has an obvious positive impact on subjective cognition.

*H2b*: Macroeconomics has a strongly positive effect on openness.

*H2c*: Macroeconomics has an appreciable positive influence on hope.

*H2d*: Macroeconomics has an apparent positive effect on tenacity.

#### Entrepreneurial PsyCap and farmers’ entrepreneurial behavior

Entrepreneurial PsyCap has a vital impact on the formation of farmers’ entrepreneurial behavior (Zhao et al., 2020). Some studies have found that entrepreneurial PsyCap has a synergistic and interactive effect on farmers’ entrepreneurial behavior (de Lima et al., 2020). Moreover, it has a more remarkable influence on entrepreneurs’ perception of entrepreneurial opportunities and entrepreneurial environment than social capital (Yalap et al., 2020). Entrepreneurial sustainability relies heavily on positive entrepreneurial PsyCap. According to it, the following hypotheses are proposed:

*H3a*: Subjective cognition has a distinctly positive effect on the formation of farmers' entrepreneurial behavior.

*H3b*: Open-mindedness has a prominent positive impact on the formation of farmers' entrepreneurial behavior.

*H3c*: Hope has an apparent positive effect on the formation of farmers' entrepreneurial behavior.

*H3d*: Tenacity has a strongly positive influence on the formation of farmers' entrepreneurial behavior.

**Figure 2:**
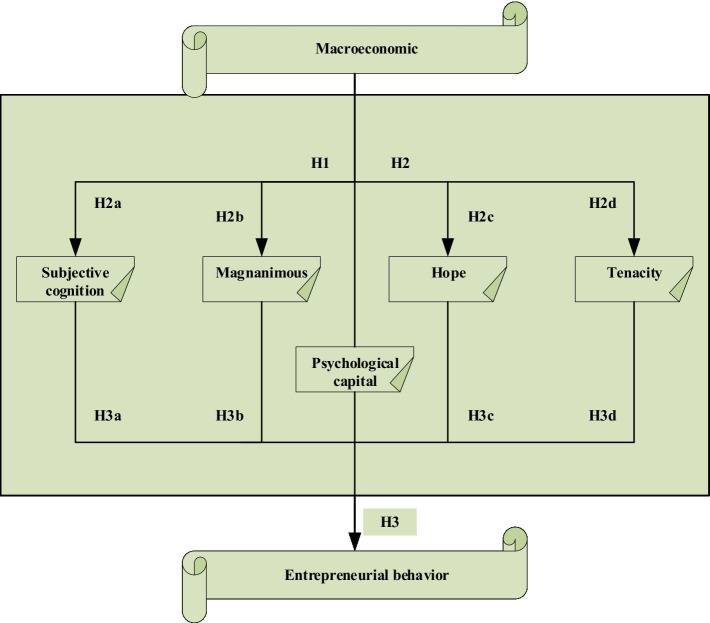
Conceptual model diagram of macroeconomics, entrepreneurial PsyCap, and entrepreneurial behavior.

Based on the above research assumptions, a research conceptual model is proposed, as shown in Figure 2.

### Questionnaire design and data processing

#### Scale selection and questionnaire design

**Figure 3:**
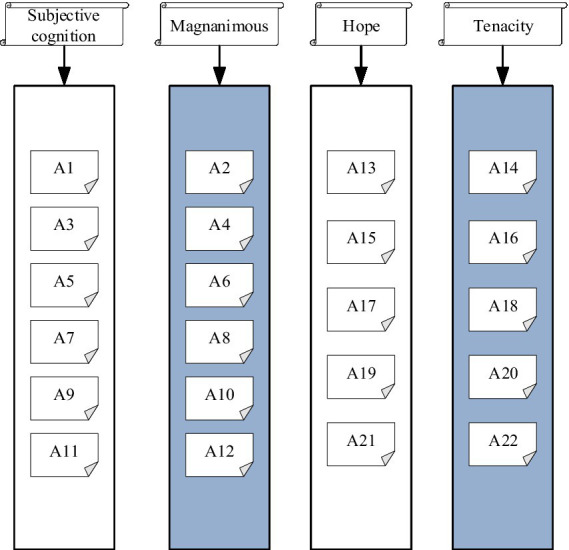
Distribution of items on the entrepreneurial PsyCap scale.

For the measurement of entrepreneurial PsyCap and macroeconomics, the existing mature scales (Roopesh, 2020) are improved and revised in combination with research directions. Among them, the entrepreneurial PsyCap scale mainly holds 22 indicators in four dimensions: subjective cognition, open-mindedness, hope, and tenacity; the macroeconomic scale mainly contains 6 indicators, and the entrepreneurial behavior includes 6 indicators. Variables are measured using a 5-point Likert scale, with a minimum of 1, indicating “very disagree,” and a maximum of 5, indicating “very agree” (Lvarez et al., 2020). The index distribution of the entrepreneurial PsyCap scale is demonstrated in Figure 3.

#### Data collection

This research collected data by means of a questionnaire. Five village groups in Shandong Province, China were selected as the survey objects. The method was a random sampling survey. A total of 200 questionnaires were distributed. The questionnaires were released through a combination of the questionnaire star applet and offline distribution, and the data were sorted and analyzed to ensure authenticity and reliability of the data. The questionnaires were distributed for 1 week, and 185 valid questionnaires were finally recovered, with an effective recovery rate of 92.5%. The above data illustrates that the questionnaire survey set up this time meets the requirements and can be used for research. The age range of the people participating in the questionnaire survey is from 20 to 60 years old. The reasons for selecting this age group are: on the one hand, there are certain differences and diversity in the thinking of people of different ages in entrepreneurship. On the other hand, it is to make the results of the questionnaire survey more complete, the data is more comprehensive and more convincing. The survey results declare that there are 101 male farmers and 84 female farmers in this research. 30 farmers in the age group of 20–30 years old, and 40 farmers in the age group of 30–40 years old. There are 50 farmers aged 40–50, and 65 farmers aged 50–60. 35 farmers belonging to poor families, 150 farmers not belonging to poor families. A total of 53 farmers belong to one-child farmers, and 132 farmers do not belong to one-child households. 125 farmers have no debts, 35 farmers have household debts ≤30,000, and 25 farmers have household debts >30,000.

#### Reliability and validity analysis of the questionnaire

The reliability and validity of the questionnaire were tested by the Cronbach coefficient and Kaiser-Meyer-Olkin (KMO) method, respectively. After the test, it was found that the reliability and validity of the questionnaire were higher than the standard value, and the results were good, indicating that the set questionnaire had certain reliability.

#### Data processing and scale testing

As a commonly used data statistical analysis software, SPSS software has the advantages of high data accuracy and fast calculation speed. Therefore, the original data of this model is the questionnaire survey data, and the valid questionnaires are sorted and encoded into the Statistical Product and Service Solutions (SPSS) 26.0 software, and archived. When processing data, each option is coded as 1–5 in turn, and SPSS and Analysis of Moment Structure (AMOS) 19.0 software are used for data analysis. The designed data analysis methods include basic descriptive statistics, analysis of variance, sample t-test, data robustness analysis, and path test.

## Empirical analysis and data inspection

### Analysis of the status quo of farmers’ entrepreneurial PsyCap

**Figure 4:**
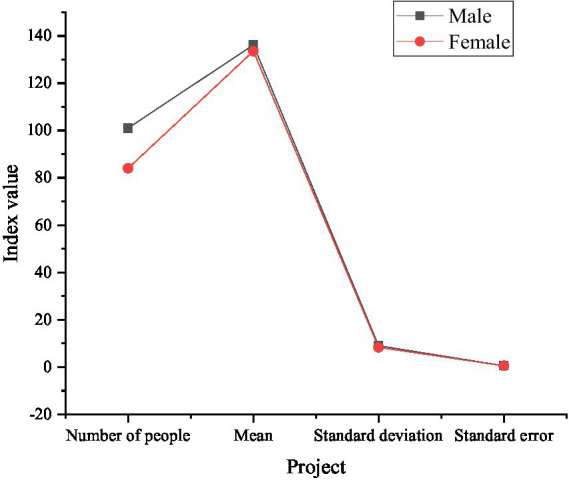
The descriptive statistics of gender and age on entrepreneurial PsyCap.

The descriptive statistics of gender and age on entrepreneurial PsyCap are presented in Figure 4.

**Figure 5:**
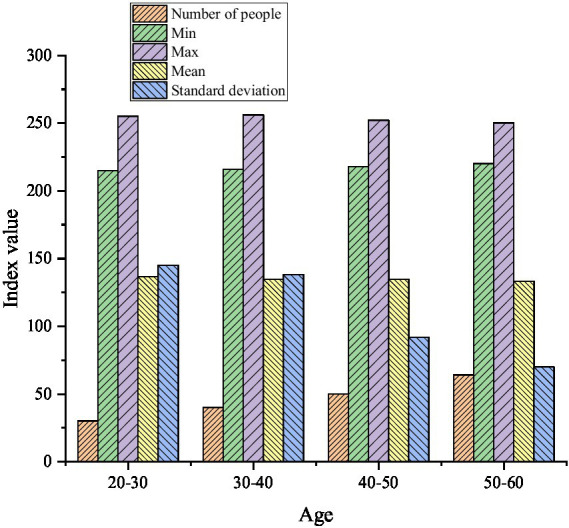
The descriptive statistics on the entrepreneurial PsyCap of farmers at different ages.

In Figure 4, the average score of entrepreneurial PsyCap of male farmers is 136.20; the average score of female farmers is 133.56, and the average score of entrepreneurial PsyCap of men is higher than that of women. Figure 5 indicates the descriptive statistics on the entrepreneurial PsyCap of farmers at different ages.

**Figure 6:**
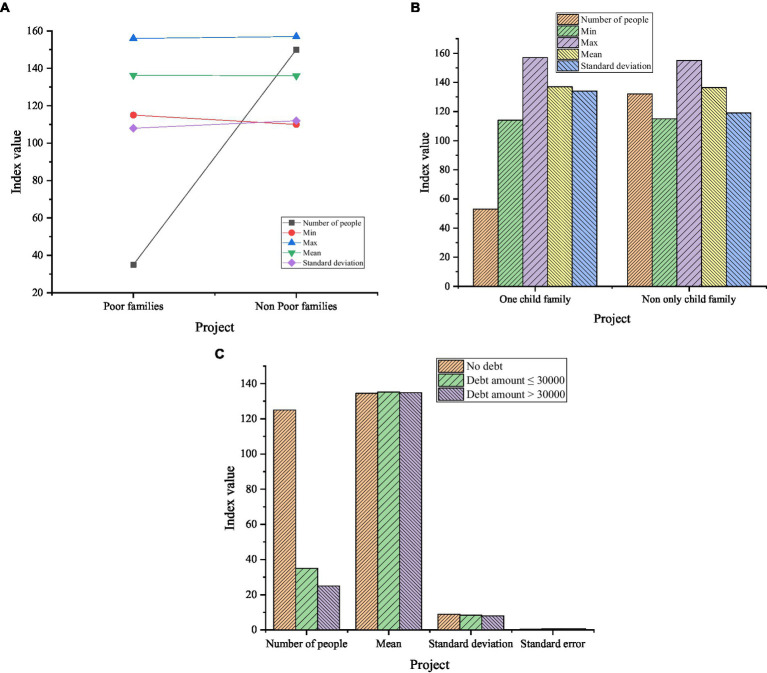
Descriptive statistics on entrepreneurial PsyCap of households who are poor families, whether they are one-child families, and different household debt situations. **(A)** Descriptive statistics of entrepreneurial PsyCap of poor families; **(B)** Descriptive statistics of entrepreneurial PsyCap of one-child families; **(C)** Descriptive statistics of entrepreneurial PsyCap of households with different household debts.

Figure 5 manifests that the average scores of entrepreneurial PsyCap of farmers in different age groups are 136.60, 134.78, 134.84, and 133.27, respectively. It can be seen that among the surveyed farmers, the average score of entrepreneurial PsyCap of the 20-30-year-old is the highest, and the lowest average score is the 50-60-year-old farmers. Figure 6 reveals the descriptive statistical results of the entrepreneurial PsyCap of the farmers whether they are poor families, whether they are one-child families, and with different household debt situations.

Figure 6 details that the average score of farmers’ entrepreneurial PsyCap of poor family is higher than that of non-poor family; the average score of entrepreneurial PsyCap of the non-one-child family is 136.37, and the score of the one-child family is 136.95. The entrepreneurial PsyCap of the one-child families is higher than that of the non-one-child families. The average scores of farmers’ entrepreneurial behavior with different household debts are 134.86, 135.48, and 135.14, respectively. It means that the average score of farmers’ entrepreneurial behavior whose household debt is less than 30,000 is the highest, and the average score with no debt is the lowest. The above data reveal that the poverty status of the family, whether it is an only child, and the different debt status will have an impact on the entrepreneurial PsyCap of the farmers. This is because the different family statuses will have an influence on the input and output of the family economy. Generally speaking, if a family has excess funds in storage, the PsyCap of farmers will be enhanced, and they will tend to have the idea of starting a business.

### Analysis of the status quo of farmers’ entrepreneurship behavior

#### The relationship between each indicator and entrepreneurial behavior

**Figure 7:**
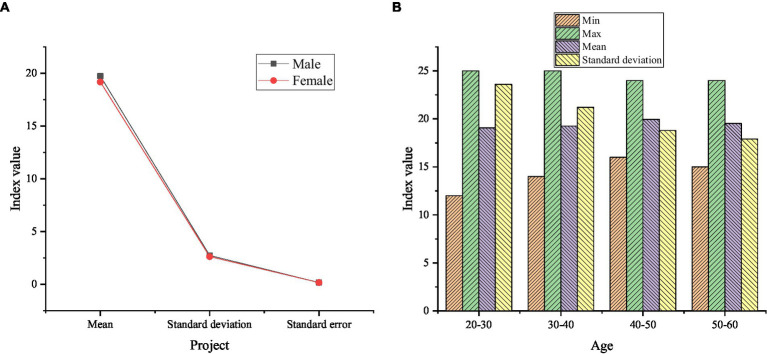
Descriptive statistics of gender and age on entrepreneurial behavior. **(A)** Descriptive statistics of entrepreneurial behavior of male and female farmers; **(B)** Descriptive statistics of entrepreneurial behavior of farmers of different age groups.

The descriptive statistics of gender and age on entrepreneurial behavior are exhibited in Figure 7.

**Figure 8:**
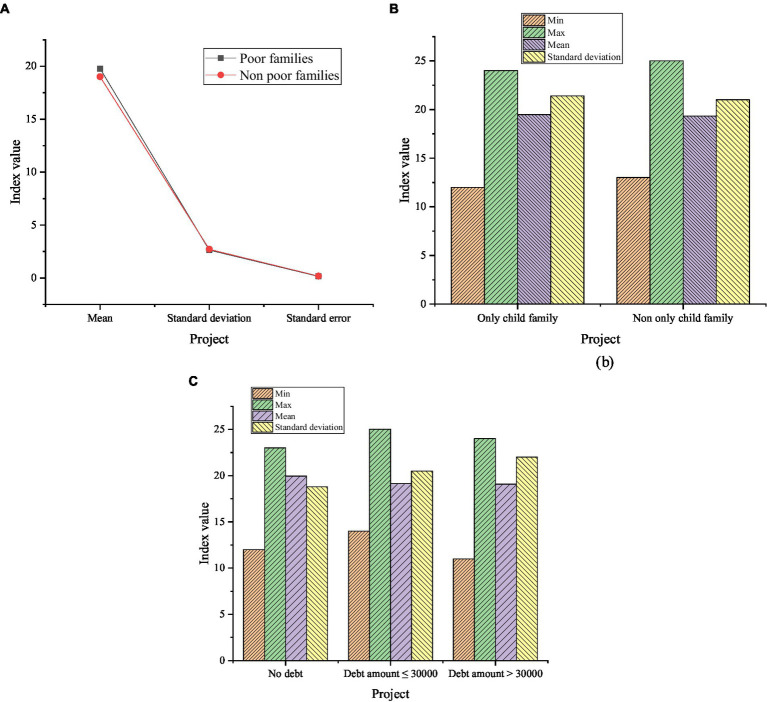
Descriptive statistics on entrepreneurial behavior of farmers whether it is a poor family, one-child family, or with different debt situations. **(A)** Descriptive statistics on entrepreneurial behavior of farmers whether it is a poor family; **(B)** Descriptive statistics on entrepreneurial behavior of farmers whether it is a one-child family; **(C)** The descriptive statistics of the entrepreneurial behavior of the farmers with different household debts.

In Figure 7, the average score for male entrepreneurial behavior is 19.72, and the average score for female entrepreneurial behavior is 19.17. The average score of male entrepreneurial behavior is higher than that of females. The average scores of farmers’ entrepreneurial behavior in different age groups are 19.05, 19.25, 19.94, and 19.52, which refers that the average score of farmers’ entrepreneurial behavior in the age group of 40–50 years old is the highest, and in the age group of 20–30 years old is the lowest. Figure 8 denotes the descriptive statistical results of farmers’ entrepreneurial behavior whether it is a poor family, one-child family, or with different debt situations.

In Figure 8, the average score of entrepreneurial behavior of farmers of the surveyed poor family is 19.78; the average score of the non-poor family is 19.01, and the average score of entrepreneurial behavior of farmers of poor families is higher than that of non-poor families. The average scores of entrepreneurial behavior of farmers of non-one-child families and one-child families are 19.33 and 19.47, respectively. The average score of entrepreneurial behavior of farmers of the one-child family is higher than that of the non-one-child family. The average scores of entrepreneurial behavior in different household debt situations are 19.93, 19.12, and 19.07. The average score of farmers’ entrepreneurial behavior with no household debt is the highest, and the average score of farmers’ entrepreneurial behavior with household debt greater than 30,000 is the lowest.

#### Significance results of each index based on sample t-test and analysis of variance

The significant results of each indicator based on the sample t-test and analysis of variance are indicated in Table 1.

**Table 1:** Significant results of each indicator on entrepreneurial behaviour based on the sample t-test.

Indicator	Gender	Age	Whether it is a poor family	Whether it is a one-child family	Household debts
Significance	0.052	0.061	0.057	0.84	0.54

Table 1 manifests that these indicators such as gender, age, whether it is a poor family, whether it is a one-child family, and household debts are all more significant than 0.05. It means that in these indicators, the differences in the entrepreneurial PsyCap of farmers are small and insignificant.

### Robustness test of scale data

Since the relationship between the related variables in the various data collected by the questionnaire is almost normal distribution, it is more appropriate to use the probit model than the Logistic model. To this end, the data are tested for robustness using the probit regression model, and the results are exhibited in Table 2.

**Table 2:** Results of data robustness test under Probit regression model.

Variable	Gender	Age	Whether it is a poor family	Whether it is a one-child family	Household debts	Observations	R^2^
Numerical value	0.40^***^ (0.05)	−0.01^***^ (0.03)	0.04^***^ (0.01)	0.05^***^ (0.04)	0.09^***^ (0.06)	7,929	0.24

Among them, “***” means very significant.

In Table 2, after inspection and analysis, the data of each indicator of the questionnaire has a small fluctuation range and high robustness. It illustrates that the establishment of the indicators under this model has certain reliability.

### Path testing for SEM

The reason for choosing the SEM is that the model can deal with multiple dependent variables at the same time, can tolerate measurement errors in independent and dependent variables, and can also estimate factor structure and factor relationships, allowing for a more elastic measurement model and estimating the fit of the overall model. The accuracy of the model is high, and the results are simple and easy to understand.

The results of the path test of SEM are displayed in Table 3.

**Table 3:** The path test of SEM.

Hypotheses	Hypothetical Path	Standard path coefficient	R^2^	Test result
H1	Macroeconomics → Entrepreneurial behavior	0.40^***^	0.16	Accept
H2a	Macroeconomics → Subjective cognition	0.59^***^	0.35	Accept
H2b	Macroeconomics → Open-minded	0.53^***^	0.28	Accept
H2c	Macroeconomics → Hope	0.43^***^	0.19	Accept
H2d	Macroeconomics →Tenacity	0.60^***^	0.36	Accept
H3a	Subjective cognition → Entrepreneurial behavior	0.30^***^	0.09	Accept
H3b	Open-minded → Entrepreneurial behavior	0.19^***^	0.03	Accept
H3c	Tenacity → Entrepreneurial behavior	0.03	0.08	Refuse
H3d	Hope → Entrepreneurial behavior	0.08	0.07	Refuse

Among them, “***” means very significant.

Table 3 displays that the software is used to perform the effect test and parameter estimation of the structural equation. The operation results signify that the factor loadings of all latent variable measurement indicators are between 0.03 and 0.35, and all reach the 0.05 significance level, indicating that the model fully meets the basic adaptation standard. This model is identifiable. Table 3 refers that the standardized path coefficient of the macroeconomic effect on the formation of farmers’ entrepreneurial behavior is 0.40, which is highly distinct (*p* < 0.01). The standardized path coefficients of the impact of subjective cognition and open-mindedness on the formation of farmers’ entrepreneurial behavior are 0.30 and 0.19, which have a very significant effect (p < 0.01). However, tenacity and hope did not have an appreciable impact on the formation of farmers’ entrepreneurial behavior.

In summary, the seven hypotheses of H1, H2a, H2b, H2c, H2d, H3a, and H3b are established, and the hypotheses H3c and H3d are not established. The path test results in Table 3 denote that entrepreneurial PsyCap has a prominent positive impact on the formation of farmers’ entrepreneurial behavior. Subjective cognition and open-mindedness can have a strongly positive effect on the formation of farmers’ entrepreneurial behavior. The macroeconomics can noticeably affect the four kinds of entrepreneurial PsyCap of farmers’ subjective cognition, open-mindedness, hope, and tenacity, and then have different degrees of positive impact on the formation of their entrepreneurial behavior. It demonstrates that entrepreneurial PsyCap plays a mediating role in the macro-economy and farmers’ entrepreneurial behavior. Macroeconomic growth has an apparent positive influence on farmers’ entrepreneurial behavior.

## Discussion

Regarding the relationship among farmers’ entrepreneurial behavior, entrepreneurial PsyCap, and macroeconomics, corresponding hypotheses are put forward according to the literature. For example, Yu and Xu (2022) observed the relationship between the economic cycle and entrepreneurial activities at the macro level, devoted themselves to exploring entrepreneurial models, and found the predictive role of some external environmental variables, and related to macroeconomic indicators. Their research results testify that the higher the macroeconomic level, the higher the entrepreneurial PsyCap of farmers, and the stronger their entrepreneurial behavior. It is the same as some of the proposed hypotheses, which have been verified. In addition, Ullah et al. (2020) believed that macroeconomics is an important external factor affecting individual entrepreneurial PsyCap, which is also reflected. However, the difference between the research and the former is that it refines the PsyCap of farmers, and studies the entrepreneurial behavior of farmers through the sub-branch of PsyCap. On the basis of previous research, the relationship between entrepreneurial PsyCap, macroeconomics, and farmers’ entrepreneurial behavior is verified. Furthermore, the research content is improved on the basis of the previous literature. The factors influencing farmers’ entrepreneurial behavior are analyzed by conducting questionnaire surveys on people of different ages. On the whole, it is a theoretical reference for reference.

## Conclusion

First, the entrepreneurial PsyCap of farmers’ entrepreneurship is analyzed. Second, the hypothesis of this work is put forward, an SEM is implemented, and a questionnaire is designed to collect relevant data. At last, through the implementation of an SEM, the relationship between farmers’ entrepreneurial behavior and macroeconomics is explored. The empirical analysis results state that there are small differences in the entrepreneurial PsyCap of farmers of diverse indicators such as age, gender, household debt, and whether is a poor family. This denotes that the entrepreneurial behavior of farmers in various situations will be different. Based on the path test of SEM, it can be obtained that macroeconomics has a prominent positive influence on entrepreneurial behavior. The disadvantage is that the research object only includes village farmers who are not determined to start a business or have not yet formed entrepreneurial awareness. There may be a situation where they have an unclear understanding of themselves and entrepreneurship, which will cause some deviations in some data. In the future, it is necessary to conduct further investigations and interviews with farmers who have had entrepreneurial experience, to continuously improve the designed model. The objective is to explore the impact of macroeconomics on farmers’ entrepreneurial behavior and the mediating role of entrepreneurial psychology in it.

## Data availability statement

The raw data supporting the conclusions of this article will be made available by the authors, without undue reservation.

## Ethics statement

The studies involving human participants were reviewed and approved by Chinese Academy of Social Sciences Ethics Committee. The patients/participants provided their written informed consent to participate in this study. Written informed consent was obtained from the individual(s) for the publication of any potentially identifiable images or data included in this article.

## Author contributions

All authors listed have made a substantial, direct, and intellectual contribution to the work, and approved it for publication.

## Conflict of interest

The authors declare that the research was conducted in the absence of any commercial or financial relationships that could be construed as a potential conflict of interest.

## Publisher’s note

All claims expressed in this article are solely those of the authors and do not necessarily represent those of their affiliated organizations, or those of the publisher, the editors and the reviewers. Any product that may be evaluated in this article, or claim that may be made by its manufacturer, is not guaranteed or endorsed by the publisher.

## References

[ref1] Anisa A. Kusnadi N. Rachmina D. (2021). Government's roles impacts the entrepreneurial orientation of rice farmers. IOP Conferen. Series: Earth Environ. Sci. 807:032072. doi: 10.1088/1755-1315/807/3/032072

[ref2] Bandyopadhyay S. Sharma A. Sahoo S. Dhavala K. Sharma P. (2021). Potential for aquifer storage and recovery (asr) in South Bihar, *India*. Sustain. For. 13:3502. doi: 10.3390/su13063502

[ref3] Can H. B. (2021). Enacted pedagogical content knowledge profiles of chemistry teachers. J. Educ. Issues 7:565. doi: 10.5296/jei.v7i1.18643

[ref4] Chen M. (2019). The impact of expatriates’ Cross-cultural adjustment on work stress and job involvement in the high-tech industry. Psychol. Forsch. 10:2228. doi: 10.3389/fpsyg.2019.02228 PMC6794360 31649581

[ref5] Chen M. Liu Q. Huang S. (2020). Environmental cost control system of manufacturing enterprises using artificial intelligence based on value chain of circular economy. Enterprise Info. Systems 5, 12–13. doi: 10.1080/17517575.2020.1856422

[ref6] Christy F. David A. Choudhary N. Kalgi N. Maheswari. (2021). Impact of psychological capacities on the work-life balance of entrepreneurs. Psychology (Savannah, Ga.) 58, 3869–3875. doi: 10.2139/ssrn.3897245

[ref7] Cross D. Swart J. (2021). Professional fluidity: reconceptualising the professional status of self-employed neo-professionals. Organ. Stud. 42, 1699–1720. doi: 10.1177/0170840620964985

[ref8] Cumming M. M. O'Brien K. M. Brunsting N. C. Bettini E. (2021). Special educators' working conditions, self-efficacy, and practices use with students with emotional/behavioral disorders. Remedial Spec. Educ. 42, 220–234. doi: 10.1177/0741932520924121

[ref9] de Lima L. G. Nassif V. M. J. Garon M. (2020). The power of psychological capital: the strength of beliefs in entrepreneurial behavior o poder do capital psicológico: a fora das crenas no comportamento empreendedor. Revista de Administração Contemporânea 24, 317–334. doi: 10.1590/1982-7849rac2020180226

[ref10] Ephrem A. N. Nguezet P. M. D. Charmant I. K. Murimbika M. E. Manyong V. (2021). Entrepreneurial motivation, psychological capital, and business success of young entrepreneurs in the drc. Sustain. For. 13:4087. doi: 10.3390/su13084087

[ref11] Feng B. Chen M. (2020). The impact of entrepreneurial passion on psychology and behavior of entrepreneurs. Psychol. Forsch. 11:1733. doi: 10.3389/fpsyg.2020.01733 PMC7385187 32793066

[ref12] Gao Q. Wu C. Wang L. Zhao X. (2020). The entrepreneur's psychological capital, creative innovation behavior, and enterprise performance. Front. Psychol. 11:1651. doi: 10.3389/fpsyg.2020.01651, PMID: 32793048 PMC7393239

[ref13] Janker J. Vesala H. T. Vesala K. M. (2021). Exploring the link between farmers' entrepreneurial identities and work wellbeing. J. Rural. Stud. 83, 117–126. doi: 10.1016/j.jrurstud.2021.02.014

[ref14] Ji Y. L. Kim G. M. Kim E. J. (2021). Predictors of entrepreneurial intention of nursing students based on theory of planned behavior. J. Multidiscip. Healthc. 14, 533–543. doi: 10.2147/JMDH.S288532 33664575 PMC7924248

[ref15] Kobba F. Nain M. S. Singh R. Mishra J. R. (2021). Determinants of entrepreneurial success: a comparative analysis of farm and non-farm sectors. Indian J. Agric. Sci. 91, 269–273.

[ref16] Lv Z. Wang L. Zhang W. (2021). An empirical study of factors influencing entrepreneurship using fuzzy logic: based on provincial panel data. J. Intell. Fuzzy Syst. 40, 8371–8377. doi: 10.3233/JIFS-189658

[ref17] Lvarez C. González D. O. Cruz Y. D. (2020). Face-to-face education with a virtual support: an experience of Honduras on covid-19 times. Revista Digital de Investigación en Docencia Universitaria 14:1229. doi: 10.19083/ridu.2020.1229

[ref18] Novanda R. R. Khaliqi M. Bakhtiar A. Amiruddin A. (2021). The impact of entrepreneurial characteristics and innovation characteristics on entrepreneurial skills in madura cattle farmers. IOP Conferen. Series: Earth Environ. Sci. 782:022026. doi: 10.1088/1755-1315/782/2/022026

[ref19] Pathak S. Goltz S. (2021). An emotional intelligence model of entrepreneurial coping strategies. Intern.l J. Entrepren. Behav. Res., ahead-of-print (ahead-of-print) 27, 911–943. doi: 10.1108/IJEBR-01-2020-0017

[ref20] Rogoza R. Żemojtel-Piotrowska M. Kwiatkowska M. (2018). The bright, the dark, and the blue face of narcissism: the Spectrum of narcissism in its relations to the metatraits of personality, self-esteem, and the nomological network of shyness, loneliness, and empathy. Front. Psychol. 9:343. doi: 10.3389/fpsyg.2018.00343, PMID: 29593627 PMC5861199

[ref21] Roopesh B. N. (2020). Vineland social maturity scale: an update on administration and scoring. Indian J. Clin. Psychol. 46, 91–102.

[ref22] Rosli F. Bakar T. Hamzah N. M. Young L. J. Mahshar M. (2021). Participation towards agro-entrepreneur among risda rubber smallholders in Kelantan. IOP Conferen. Series: Earth Environ. Sci. 756:012026. doi: 10.1088/1755-1315/756/1/012026

[ref23] Santoro G. Messeni-Petruzzelli A. Giudice M. D. (2020). Searching for tenacity: the impact of employee-level and entrepreneur-level tenacity on firm performance in small family firms. Small Bus. Econ. 34, 1–17. doi: 10.1007/s11187-020-00319-x

[ref24] Schneider K. (2022). Influences on the entrepreneurial activities of women academics. Psychology 13, 78–88. doi: 10.4236/psych.2022.131006

[ref25] Schneider K. Saeed V. (2021). Entrepreneurial career probabilities of adolescents. Open Psychol. J. 14, 104–112. doi: 10.2174/1874350102114010104

[ref26] Shi B. Wang T. (2021). Analysis of entrepreneurial motivation on entrepreneurial psychology in the context of transition economy. Front. Psychol. 12:680296. doi: 10.3389/fpsyg.2021.680296, PMID: 34456794 PMC8385207

[ref27] Tma B. Mbt A. Ps A. Ym B. (2020). The influence of social capital and entrepreneurial attitude orientation on entrepreneurial intentions: the mediating role of psychological capital. Eur. Res. Manag. Bus. Econ. 26, 33–39. doi: 10.1016/j.iedeen.2019.12.005

[ref28] Ullah A. Chen P. Ullah S. Zaman M. Hashmi S. H. (2020). The nexus between capital structure, firm-specific factors, macroeconomic factors and financial performance in the textile sector of Pakistan. Heliyon 6, e04741–e04710. doi: 10.1016/j.heliyon.2020.e04741 32895635 PMC7456801

[ref29] Wijaya B. S. Sutawidjaya A. H. Syaiful M. (2020). Changing the mindset in the culinary business environment: from entrepreneur to branderpreneur. IOP Conferen. Series Earth Environ. Sci. 469:012045. doi: 10.1088/1755-1315/469/1/012045

[ref30] Yalap O. Ylmaz H. Polat S. (2020). Do psychological capital and communication skills affect entrepreneurial intention. Global J. Business Econ. Manag. Current Issues 10, 21–30. doi: 10.18844/gjbem.v10i1.4540

[ref31] Yamamura S. Lassalle P. (2022). Extending mixed embeddedness to a multi-dimensional concept of transnational entrepreneurship. Comparative Migration Stud. 10:43. doi: 10.1186/s40878-022-00288-y

[ref32] Yang S. Wang H. Wang Z. Koondhar M. A. Ji L. Kong R. (2021). The nexus between formal credit and e-commerce utilization of entrepreneurial farmers in rural China: a mediation analysis. J. Theor. Appl. Electron. Commer. Res. 16, 900–921. doi: 10.3390/jtaer16040051

[ref33] Yu L. Xu W. (2022). Institutional conformity, entrepreneurial governance and local contingency: problematizing central-local dynamics in localizing china's low-income housing policy. Environ. Plann. Economy Space 54, 508–532. doi: 10.1177/0308518X211061400

[ref34] Yueh H. P. Wu Y. J. Chen W. F. (2020). Editorial: the psychology and education of entrepreneurial development. Front. Psychol. 11:27. doi: 10.3389/fpsyg.2020.00027, PMID: 32038440 PMC6992604

[ref35] Zhao J. Wei G. Chen K. H. Yien J. M. (2020). Psychological capital and university students' entrepreneurial intention in China: mediation effect of entrepreneurial capitals. Front. Psychol. 10:2984. doi: 10.3389/fpsyg.2019.02984, PMID: 32038375 PMC6989491

[ref36] Zhong H. Yan R. Li S. Chen M. (2020). The psychological expectation of new project income under the influence of the Entrepreneur’s sentiment from the perspective of information asymmetry. Psychol. Forsch. 11:1416. doi: 10.3389/fpsyg.2020.01416 PMC7381333 32774311

